# Economic evaluation of a complex intervention to improve the mental health of maltreated children in foster care (BeST? Services trial)

**DOI:** 10.1093/pubmed/fdaf038

**Published:** 2025-04-06

**Authors:** Manuela Deidda, Helen Minnis, Karen Crawford, Robin Young, Gary Kainth, Julia Donaldson, Matt Forde, Alex McConnachie, Christopher Gillberg, Marion Henderson, Philip Wilson, Kathleen A Boyd, Emma McIntosh

**Affiliations:** Health Economics and Health Technology assessment, School of Health and Wellbeing, Clarice Pears Building, 90 Byres road G12 8TB, University of Glasgow, Glasgow, UK; Centre for Developmental Adversity and Resilience (CeDAR), Mental Health and Wellbeing, School of Health and Wellbeing, Clarice Pears Building, 90 Byres road, G12 8TB University of Glasgow, Glasgow, UK; Centre for Developmental Adversity and Resilience (CeDAR), Mental Health and Wellbeing, School of Health and Wellbeing, Clarice Pears Building, 90 Byres road, G12 8TB University of Glasgow, Glasgow, UK; Robertson Centre for Biostatistics, School of Health and Wellbeing Clarice Pears Building, 90 Byres Road, Glasgow, G12 8TB University of Glasgow, Glasgow, UK; Centre for Developmental Adversity and Resilience (CeDAR), Mental Health and Wellbeing, School of Health and Wellbeing, Clarice Pears Building, 90 Byres road, G12 8TB University of Glasgow, Glasgow, UK; Glasgow Infant and Family Team, NSPCC, Pavillion 2 Rowan Business Park Ardlaw Street Glasgow G51 3RR Glasgow, UK; Glasgow Infant and Family Team, NSPCC, Pavillion 2 Rowan Business Park Ardlaw Street Glasgow G51 3RR Glasgow, UK; Robertson Centre for Biostatistics, School of Health and Wellbeing Clarice Pears Building, 90 Byres Road, Glasgow, G12 8TB University of Glasgow, Glasgow, UK; Centre for Developmental Adversity and Resilience (CeDAR), Mental Health and Wellbeing, School of Health and Wellbeing, Clarice Pears Building, 90 Byres road, G12 8TB University of Glasgow, Glasgow, UK; The Gillberg Neuropsychiatry Centre and Institute of Neuroscience and Physiology University of Gothenburg Gillbergcentrum Kungsgatan 12, vån 2411 19 Göteborg; MRC/CSO Social and Public Health Sciences Unit, School of Health and Wellbeing, Clarice Pears Building, 90 Byres Road, Glasgow, G12 8TB University of Glasgow, Glasgow, UK; School of Social Work and Social Policy, University of Strathclyde, 141 St James Road G4 0LT Glasgow, UK; Institute of Applied Health Sciences, University of Aberdeen Aberdeen, AB24 3FX; Department of General Practice, Institute of Public Health Science, Department of Public Health, Section of General Practice University of Copenhagen Øster Farimagsgade 5 opg. Q1353 Copenhagen; Health Economics and Health Technology assessment, School of Health and Wellbeing, Clarice Pears Building, 90 Byres road G12 8TB, University of Glasgow, Glasgow, UK; Health Economics and Health Technology assessment, School of Health and Wellbeing, Clarice Pears Building, 90 Byres road G12 8TB, University of Glasgow, Glasgow, UK

**Keywords:** children, cost effectiveness, mental health

## Abstract

**Background:**

Children in foster care who have experienced abuse and neglect are at risk of poor long-term health and societal outcomes. Evidence on the costs, benefits and cost-effectiveness of early interventions aimed at improving the mental health of abused and neglected children is limited.

**Methods:**

This study reports the within-trial economic evaluation alongside BEST?, a randomized controlled trial comparing the New Orleans Intervention Model (NIM) with services as usual (SAU), targeting children aged 0–60 months entering UK foster care.

In line with guidance for conducting economic evaluations of complex and social care interventions, a cost-utility analysis (CUA) estimated incremental cost of NIM per quality-adjusted life year (QALY); a cost-effectiveness analysis estimated incremental cost per unit improvement in child mental health; and a cost-consequence analysis combined costs with broad-ranging outcomes.

**Results:**

NIM is significantly more costly than SAU (NIM: £10 002; SAU: £4336), with wide cost variations according to context. There are no significant differences between NIM and SAU in QALYs or child mental health.

**Conclusions:**

Within the current UK care systems, NIM is not a cost-effective alternative to SAU. However, these results need to be interpreted with caution and within the prevailing service provision context.

## Introduction

Abuse and neglect in childhood is associated with significantly detrimental health and social outcomes over the life course, including mental and physical illness, disability,^1^ poor quality of life,^2^ lower education and employment outcomes^3^ and crime, placing a major economic burden on the health and social care systems, individuals, families, and society more broadly. Preventing maltreatment could lead to estimated lifetime cost savings of over £89 000 per maltreated child, with major cost drivers in terms of reduced employment (£14 037), social care (£38 132) and short-term mental (£18 553) and physical (£18 673) health.^4^

Children moving into foster care who have experienced abuse and neglect, are further exposed to additional trauma caused by separation from birth families and placement breakdowns,^5^^,^^6^ with potentially devastating consequences in terms of emotional and developmental problems that can persist to adulthood in the absence of any intervention. Globally, there are 2.7 million looked-after children, with 105 400 in the UK, where this study was conducted.^7^ This figure continues to grow, supporting the argument for investment in evidence-based interventions to improve the mental health and societal outcomes for this population. Failures in the care system may also be associated with increased costs because of placement instability and returns to care.^8^ Given the huge societal cost burden associated with maltreatment, finding cost-effective interventions to mitigate its adverse consequences is a public health priority.^9^ Interventions targeting children’s health and wellbeing have the potential to be cost-effective, with potentially long-lasting impacts on physical and mental health, with consequent positive societal effects in terms of better education and employment opportunities, less involvement in crime, and less welfare dependency.^10^ However, evidence on the effectiveness (in terms of emotional, developmental, and relational outcomes) of interventions targeting pre-school children in foster care is mixed^11^^,^^12^, and evidence on cost-effectiveness is scarce. The BeST? study fills this gap by evaluating an intensive intervention (New Orleans Intervention Model, NIM) targeting children in foster care with experience of abuse and neglect against services-as-usual (SAU) in the UK.^13^

This paper details the methods and results of the within-trial economic evaluation of BeST?. The analysis has been conducted following the latest guidance for conducting economic evaluations of complex public health interventions,^14–16^ as well as NICE social care guidance.^17^

## Methods

### The BeST? trial

BeST? is a multi-site RCT with a nested economic evaluation, conducted in two sites in the UK: Greater Glasgow and Clyde (comprising Glasgow City Council and Renfrewshire Council) and London (including six boroughs: Croydon, Tower Hamlets, Sutton, Bromley, Barking and Dagenham). Children aged 0–60 months entering care for reasons associated with maltreatment were randomized into two groups: NIM and SAU. Recruitment took place between January 2012 and July 2021.

NIM is an intensive, targeted, individualized, family-based intervention that aims to offer assessment and trial-of-treatment for the birth family and make timely recommendations for rehabilitation back to the birth home or adoption.^18^^,^^19^ The intervention is delivered by a multidisciplinary team including mental health specialists (psychologist, psychiatrist) and social workers. SAU is social work services, where children are usually allocated to social workers providing assessments of parental capacity and signposting to specific services. SAU differs between sites: in Glasgow parental assessments are carried out by a specialist team of social workers, whereas in most of the London boroughs specialist assessments are either carried out by independent social workers (ISWs), psychologist or psychiatrist or the allocated social worked can engage additional services if required. Further details of the BeST study and the main study outcomes are reported elsewhere.^13^^,^^20^

### Economic evaluation frameworks

Following updated NICE guidance,^16^ the main economic evaluation framework was a cost-utility analysis (CUA), combining the total cost of the intervention and control with quality-adjusted life years (QALYs). Results were reported in terms of the incremental cost per additional QALY generated by the intervention. A secondary analysis used the change in the primary outcome strengths and difficulties questionnaire (SDQ) that occurred between baseline and 2.5 years as an outcome measure in a cost-effectiveness analysis (CEA) framework. In consideration of the complexity of this public health intervention,^14^^,^^21^ we used a cost-consequence analysis framework to generate evidence on the multi-sectoral outcomes (e.g. time to permanent placement; PIRGAS score; etc.) and costs/savings generated by the intervention. The NHS&PSS perspective was used in the base-case analysis. The public sector perspective, including contacts with the police and admission to residential or respite care in addition to usage of NHS and social service, and a broader, societal perspective (including the cost of childcare) were used as sensitivity analyses. A comparison of cost differences between sites was also conducted.

### Identifying, measuring, and valuing costs and outcomes

Individual-level resource use data were collected prospectively within-trial at all the three relevant time points (baseline, 1 year and 2.5 year after baseline). Information on additional service use (ASU, i.e. number of contacts for several different services such as hospital admissions, police contacts, day care or nursery usage, etc.) beyond those provided directly through the services has been collected by means of a questionnaire at each point of follow-up. Unit cost information was collected from routine sources such as the Personal Social Services Resource Unit, NHS Reference costs^4^^,^^5^ or secondary literature. Costs for each component of resource use was expressed in pounds sterling (£) for cost year 2020/2021. Supplementary Appendix S1 provides a detailed description of the cost of the intervention and control and ASU.

Children’s quality of life at three time points (baseline, 1 year and 2.5 years) was captured using the PedsQL questionnaire. As a preference-based index for the PedsQL is currently not available, PedsQL scores were mapped to CHU9D utilities using the algorithm developed by Kelly et al.^22^ facilitating calculation of QALYs. QALYs for each participant were calculated as the area under the curve following the trapezium rule, which assumes linear interpolation between follow-up points.^23^^,^^24^

### Economic analysis methods

Incremental mean QALYs and costs between treatment groups were estimated using generalized linear model (GLM) regression, adjusting for baseline covariates.^25^ The primary analysis was an intention to treat (ITT). A secondary analysis estimated the complier average causal effect (CACE),^26^ to account for three types of deviation from the planned treatment assignment: (i) participants randomized to GIFT/LIFT but receiving services as usual (SAU); (ii) no-engagers, i.e. GIFT/LIFT participants not attending the scheduled assessments and appointments; and (iii) participants originally randomized to GIFT/LIFT, who were placed on a waiting list and were only offered consultation. Two incremental cost-effectiveness ratios (ICERs) were calculated to evaluate the incremental cost per QALY (which will be calculated from PedsQL scores) and the incremental cost per improvement in SDQ (the primary outcome from the trial). All analyses were performed using Stata version 17.

Uncertainty surrounding the estimate of incremental costs, QALYs, and ICERs was investigated by a nonparametric bootstrap of the cost and effect pairs for 1000 iterations and presented on the cost-effectiveness plane with a 95% confidence interval of the bootstrapped ICER estimated. Results were summarized using a cost-effectiveness acceptability curve to reflect the probability of NIM being cost-effective at various willingness-to-pay thresholds (£20 000 and £30 000/QALY threshold).^16^

Missing costs of NIM and SAU were estimated using deterministic mean imputation, by trial arm and site. Multiple imputation procedures using chained equations were used to impute follow-up missing data separately for each arm of the trial, creating 60 imputed datasets.^27^ Predictive mean matching was used in order to deal with non-normality of cost and outcome data.^28^

## Results

### Missing data

Supplementary Table 2 shows details on percentage of missing data, by intervention arm, for baseline characteristics and cost categories and outcomes (PedsQL and SDQ) at each time point.

Complete-case analysis consisted of the children with completed data on baseline characteristics, who completed all PedsQL profiles and resource use data at each time point (baseline, 1 year, 2.5 years).

As total costs and total QALYs are cumulative quantities, any missing data at baseline or any of the follow-up points results in those children’s data being removed from the complete-case analysis. As total QALY and total costs have been adjusted for baseline characteristics, those patients with missing values in any of the covariates which have been used in the regression have been removed. This results in 106 participants in the complete-case analysis (61 in the SAU arm; 45 in the NIM arm), representing a 75% overall missingness rate. Following best practice,^29^ we conducted sensitivity analyses using different imputation models, but results were not sensitive to the model used (results not reported, but available upon request). Descriptive statistics are reported for costs and outcomes considering available case scenarios (Supplementary Tables 4–6). Given the amount of missing data detected in the trial, the base-case CUA and CEA analysis was performed in the multiple imputed dataset, while a sensitivity analysis was also performed in the complete-case dataset.

### Cost of the intervention

Table 1 shows the mean total cost/child of NIM and SAU, for each site. NIM is more expensive than SAU (£10 002 vs. £4336) as would be expected given the intensive nature of the NIM intervention. Considering the cost by site, London costs less than Glasgow for both SAU (£2782 vs. £4724 respectively) and NIM intervention (£6104 vs. £11 514 respectively).

**Table 1:** Mean cost per child of NIM and SAU per site and total, GBP (£), 2021

Cost of SAU intervention, by site (£)					
Site	Obs	Mean	SD	Min	Max
Total (Glasgow + London)	195	**4336**	5795	0	35 269
Glasgow	156	**4724**	6319	0	35 269
London	39	**2782**	2351	0	8680
*Cost of NIM intervention, by site (£)*					
*Site*	*Obs*	*Mean*	*Std. dev.*	*Min*	*Max*
Total (Glasgow + London)	136	**10 002**	9896	0	47 923
Glasgow	98	**11 514**	10 313	126	47 923
London	38	**6104**	7538	0	36 427

Descriptive statistics on available-case analysis (i.e. dataset with non-missing information on CM and NIM cost). Obs = observations, SD: standard deviation; Min: minimum, Max: maximum

**Figure 1:**
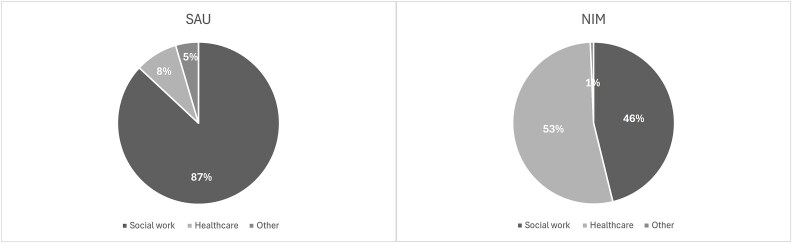
Total cost per arm, by cost type.

Figure 1 shows the breakdown of total cost, by cost type (cost of social workers; cost of healthcare workers; cost of other staff involved in the NIM and SAU assessments). The highest cost component in SAU is the cost of social workers, accounting for 87% of total cost. In NIM, costs are almost equally split between social workers and healthcare workers (46% social workers; 53% healthcare workers).

As assessments in London were carried out by ISWs, the cost of SAU in London was estimated using the national rate of £33/hour.^30^ However, ISW commands a much higher rate above the minimum national rate. In a sensitivity analysis, we explored the impact of uncertainty around the mean cost of parenting assessments by generating a cost distribution around the mean. Assuming a gamma distribution and a variance of the fee charged by ISWs equal to the national rate of £33/hour, the cost of SAU in London increases from £2782 to £3504, closing the cost difference between NIM and SAU.

### Cost-effectiveness

Supplementary Appendix S3 presents summary statistics for ASU cost and outcomes (CHU9D, SDQ total score, and QALY) for the complete case analysis and the multiply imputed dataset.

Table 2 details the incremental mean costs and QALYs for the base-case analysis (NHS & PSS perspective, including cost of intervention and control only), and then for the following scenarios, considering the cost of ASU pertaining to different perspectives in addition to the cost of intervention and control: (i) NHS & PSS perspective; (ii) public sector perspective; and (iii) societal perspective.

**Table 2:** Cost-effectiveness analysis

Cost-utility analysis (Incremental cost/incremental QALY)						
	NIM	SAU	Difference	Boostrapped CI		ICER
QALY	2.288	2.2904	**−0.0024**	*−0.0102*	*0.0081*	SAU dominates
Base-case analysis: NHS&PSS perspective cost (£): cost of intervention/control	10 360	4253	**6107**	*4971*	*7424*	SAU dominates
Scenario 1: NHS & PSS perspective cost (£): cost of intervention/control + ASU	13 271	7440	**5831**	*4437*	*7503*	SAU dominates
Scenario 2: public sector perspective cost (£): cost of intervention/control + ASU	14 062	7643	**6419**	*4933*	*7976*	SAU dominates
Scenario 3: societal perspective: cost (£): cost of intervention/control + ASU	22 046	17 274	**4772**	*2846*	*6745*	SAU dominates
*Cost-effectiveness analysis (Incremental cost/Incremental SDQ difference)*						
	*NIM*	*SAU*	*Difference*	*Boostrapped CI*		*ICER*
SDQ T3–SDQ T1	−0.3788	−1.1644	**0.7856**	*−1.9727*	*0.3290*	SAU dominates
Cost: cost of intervention/control	10 360	4253	**6107**	*4971*	*7424*	SAU dominates

Incremental outcomes and costs were analyzed using GLM regression, to account for non-normality of costs and outcomes. The modified Park test was employed to identify the optimal family, while a battery of tests (Pearson correlation tests, Pregibon link test, and modified Hosmer and Lemeshow test) were used to guide the choice of the best family. All regressions were adjusted by baseline costs and quality of life (i.e. CHU9D score at baseline), and standard errors have been adjusted by clustering at family level.

As shown in Table 2, in the base-case analysis when controlling for baseline covariates, participants randomized to NIM accrued incremental costs of £6107, and a statistically not significant decrement in quality of life of 0.0022 compared to participants randomized to the SAU arm. Sensitivity analyses considering different costing scenarios show significantly higher costs in the NIM arm as compared to SAU. Supplementary Table 6 shows the incremental cost and outcome (expressed as difference in SDQ score between Time 2 and baseline) in the two trial arms. With lower SDQ scores reflecting better mental health,^31^ we observe an improvement in SDQ between baseline and the last follow-up (2.5 years). However, participants randomized to the SAU arm experienced a better (although not statistically significant) improvement in SDQ scores than those randomized to NIM.

Supplementary Table 10 shows CUA and CEA results in the complete-case analysis, showing similar results to the multiple imputation case. The uncertainty around the incremental cost-effectiveness pair is represented on the cost-effectiveness plane (Supplementary Fig. 1). The CACE analysis confirms ITT results (results in Supplementary Appendix S5).

### Cost-consequence analysis

Supplementary Table 6 shows the cost-consequence balance-sheet, with outcomes and costs for both trial arms. Specifically, the table shows the cost of intervention and total costs (according to the different perspectives used), as well as the change in outcome occurred between baseline and T2, for both trial arms. As Table 3 shows the only significant difference between arms is in the cost of intervention vs. control. There is a small and not statistically significant difference between arms in terms of ASU (NHS & PSS perspective) being slightly lower in the NIM arm rather than in the SAU arm. No significant difference was also observed in the NIM arms when considering the emotional signalling scale, the relationship problems questionnaire, disturbance of attachment interview (DAI)-nonattachment/disinhibited and the DAI-non attachment/indiscriminate scores.

**Table 3:** Cost-consequence analysis

COSTS			
Variable *Average cost, by arm*	SAU	NIM	Improvement?
Cost of NIM vs. SAU (£)	4336	10 002	NO^*^
Additional service use NHSPSS perspective (£)	3193	2898	YES
Additional service use Public sector perspective (£)	3054	3206	NO
Additional service use Societal (£)	12 794	11 768	YES
OUTCOMES			
Variable *Difference between value at 2.5 years and value at baseline*	*SAU*	*NIM*	*Improvement?*
CHU9D	−0.004	−0.008	NO
SDQ	−2.124	0.042	NO
PIR-GAS	6.116	1.672	NO
Emotional signalling scale	3.334	4.085	YES
RPQ	−1.086	−1.494	YES
This is my baby (TIMB) acceptance score	0.389	0.369	NO
TIMB commitment score	0.710	0.634	NO
TIMB awareness score	0.435	0.336	NO
DAI-non attachment/inhibited	−0.213	0.008	NO
DAI-non attachment/disinhibited	−0.628	−0.983	YES
DAI-non attachment/indiscriminate	−0.844	−1.160	YES
DAI-non attachment/secure-base distortion	−0.879	−0.489	NO
Days from consent to permanence decision	463	551	NO
Days from consent to permanent placement	1082	1099	NO

Note: CHU9D: Child health utility instrument; PIR-GAS: parent-infant relationship; TIMB: this is my baby; DAI: disturbances of attachment interview. Average values calculated in a multiple-imputed dataset including all cost variables, CHU9D scores and secondary outcomes. Cost variables are presented as average cost, by arm. Outcome variables are presented as difference in outcome between 2.5 years and baseline. ^*^ indicates significance, *P* < .1

## Discussion

### Main finding of this study

The economic evaluation revealed no significant difference between arms in terms of outcomes (quality of life and SDQ). As anticipated, the longer timescale for delivery, high up-front treatment and prevention focus of NIM led to a statistically significantly higher cost than SAU. Mental health specialists accounted for most of NIM costs, while social worker costs represented most of the SAU costs. There was a striking ‘system’ difference between sites which was reflected in the costs: the cost difference between NIM and SAU is greater in Glasgow than in London (£6790 incremental cost between arms in Glasgow, with a £3322 incremental cost between arms in London)—and NIM costs in London were similar to SAU costs in Glasgow.

### What is already known on this topic

Early interventions to promote the health and wellbeing of children can mitigate the negative consequences of child maltreatment and have long-term positive effects on their health.^10^

The NIM intervention aims to provide the best care and outcomes for maltreated children, providing support to families as soon as they need it, early permanency when appropriate and stability where permanency is not optimal or preferable.^32^ It is likely that NIM will reduce costs both over the long term, through increased likelihood that children will have higher educational achievements, have successful lives, and be less likely to be dependent on the state, and in the short run, by reducing social workers’ time, avoiding several repeated decisions due to multiple placements.^32^ However, there is no evidence on the cost-effectiveness of NIM.

### What this study adds

This is the first time the NIM intervention has been formally evaluated for cost-effectiveness within a randomized controlled trial. This economic evaluation has also reported a full characterization of the resource use and cost components of NIM and SAU.

This study has shown the importance of contextual system differences between sites in terms of legal systems and timescales for decision-making in explaining NIM cost variations. In England, all decisions about care placements are mandated by the court within a specific time frame, whereas in Scotland these are made by a Children’s Panel of lay members. While timescales for decision-making are bound to 26 weeks in England (with any extensions having to be mandated by a judge), there is no such requirement in Scotland, so the process can be considerably prolonged, raising costs but also contributing to greater placement instability.^13^^,^^33^

Also, the findings of our sensitivity analysis considering uncertainty around the ISW rate reveal scope for decreasing the cost of NIM in London by integrating decision-making into a more efficient legal system.

### Limitations of this study

The study has been characterized by logistical challenges in data collection and extraction between sites and treatment arms. The cost of the NIM intervention was directly estimated in a bottom-up fashion from the resource use data in the National Society for the Prevention of Cruelty to Children Phoenix automated system, providing detailed information on staff and duration for each appointment. This was also possible in SAU Glasgow, where a tailored data collection instrument was designed to extract relevant information on SAU contacts from social workers’ case notes. In London, however, detailed information on appointment attendance and duration was not available; therefore, we used an average estimate by assessment type (expert opinion), conducting a probabilistic sensitivity to assess the impact of uncertainty, and generating a cost distribution around the mean.

When BeST? was designed, SDQ and PedsQL were chosen as the preferred instruments to measure children’s mental health and wellbeing, respectively, as they were both well established and previously used and validated in the child population, although with few studies focusing on pre-school children. However, there is little evidence on the suitability of PedsQL and SDQ in the maltreated and neglected population, and even less evidence on their suitability for measuring outcomes of children living in foster care. These outcomes, therefore, may not adequately measure the specificity of outcomes for this population. Also, the relatively short time horizon of BeST^?^ might not be enough to capture the developmental trajectory of children in foster care.^13^ In addition, longer timescales for a permanent placement decision in Glasgow, as compared to London, might have affected the effectiveness of the NIM intervention. In Glasgow, placement decisions were often made much later (even years later) than the NIM assessment, while, in London, the tighter 26 weeks’ timescale may not have provided the context for intervention towards safe rehabilitation with the child and their parents.^34^ This may have affected the effectiveness of the intervention in terms of improvements in mental health.

## Conclusion

The evidence generated by this economic evaluation alongside the BeST? trial shows that NIM is not a cost-effective alternative to SAU within the prevailing foster care system. Based on the within-trial analysis, there is no evidence that NIM offers an improvement in infant mental health within the current system, yet it has substantially greater costs than SAU, and therefore SAU dominates NIM. This economic evaluation reveals the urgent need for legal system changes (longer timescales in England, shorter timescales with greater legal oversight in Scotland) to facilitate timely decisions about infants’ permanent placement decisions, which we expect would be associated with better outcomes for children and lower costs to society. Further research should explore the suitability of instruments to measure mental health and development in the population of children in foster care. Also, long-term studies are advocated to capture developmental trajectories in this population.

## Supplementary Material

Supplementary_R1_fdaf038

## Data Availability

The data used in the study are not publicly available and can be shared upon reasonable request.

## Notes

**Manuela Deidda,** Research Fellow

**Helen Minnis,** Professor

**Karen Crawford,** Honorary Research Fellow

**Robin Young,** Independent biostatistician

**Gary Kainth,** Lecturer

**Julia Donaldson,** Honorary Clinical Senior Lecturer

**Matt Forde,** Partnership and Development Director, NSPCC

**Alex McConnachie,** Professor

**Christopher Gillberg,** Professor

**Marion Henderson,** Professor

**Philip Wilson,** Professor

**Kathleen Boyd,** Professor

**Emma McIntosh,** Professor
