# Supplementary material for: Economic evaluation of a complex intervention to improve the mental health of maltreated children in foster care (BeST? Services trial)
Source: J Public Health (Oxf). 2025 Apr 6;47(3):478–86. doi: 10.1093/pubmed/fdaf038 (PMC12395952; doi:10.1093/pubmed/fdaf038)
Supplement: Supplementary_R1_fdaf038 [file supplementary_r1_fdaf038.docx]

**Supplementary material**

**Supplementary appendix S1:** **Identifying, measuring and valuing costs**

- **NIM and SAU Resource use measurement**

The cost of NIM and SAU was collected using two different system. Data on NIM was collected using the National Society for the Prevention of Cruelty to Children (NSPCC)’s data collection automated system. The system collected data on attendees (children, parents, foster carers. Staff), appointment date, duration and type of appointment (e.g. circle of security, consultation, contact observation).

Equation I illustrates the resource categories of the total cost of NIM and SAU: C_NHS_  (i.e. NHS costs, such as psychologist, psychiatrist etc.) C_SW_ (i.e. cost of social workers), C_OTHER_ (other staff involved, e.g. teacher, nursery staff).

(Equation I) $C_{TOT}=C_{NHS}+C_{SW}+C_{OTHER}$

For each type of appointment, an estimation of administrative time (e.g. preparation time) was estimated using expert opinion. Information on referral to external services (e.g. addiction support, women’s aid) was also provided. The cost of SAU was collected using a different system between sites. In Glasgow, data were extracted from social workers’ case notes, and pulled into a bespoke data collection instrument, capturing information on date, nature of the contact, duration, attendees, and referral to outside services.

In London, it was not possible to collect individual-level data on the SAU intervention with the same level of details of Glasgow. Therefore, we used a standard description of typical assessments (i.e. parenting; psychological, risk) in terms of attendees, duration and type of assessment to cost the SAU cost in London. A probabilistic analysis using bootstrap has then been used in a sensitivity analysis to generate a cost distribution around the mean. The cost charged to the local authority has also been used in a sensitivity analysis. The cost data used in the economic analysis cover a period up to the date the final decision was made (date of final report).

- **Additional Service Usage (ASU)**

The ASU questionnaire asks foster carers and birth parents about the number of attendances/contacts/hours for several different services (e.g. hospital admissions, police contacts, day care or nursery usage etc.) for both the child and themselves.

Equation II illustrates the two main resource categories of total cost considering the NHS/PSS perspective: (TotCost): healthcare costs (CHC) and costs related to use of social services (CS).

(Equation II) ${TotCost}_{NHSPSS}=C_{HC}+C_{S}$

Specifically, 〖${TotCost}_{NHSPSS}$includes the following cost items: visits/contacts with the following services: General Practitioner, out of hours primary care (NHS 24), Paediatrician, Dentist, hospital outpatient visits, Emergency department visit, speech and language therapist, Drugs, alcohol worker/addiction services, domestic violence service, women protection services, adult mantal health services, CAHMS, educational psychologist.

Equation III adds the cost of contacts with the police and admission to residential and respite care to${TotCost}_{NHSPSS}$, considering a public sector perspective.

(Equation III) ${TotCost}_{PSect}={TotCost}_{NHSPSS}+ C_{police}+C_{ResCare}$

Equation IV adds the cost of childcare to ${TotCost}_{PSect}$, considering a societal perspective.

(Equation IV) ${TotCost}_{Societal}={TotCost}_{PSect}+ C_{Ccare}$

All partial costs were aggregated across post-intervention and follow up periods in order to estimate total participant costs within each arm, and the mean cost per participant per arm. The difference in average costs (and significance) between the two trial arms was estimated using regression techniques.

Where costs are accumulated and aggregated over multiple years a discounting rate of 1.5 per cent was applied per year as per NICE’s public health guidance[6].

- **Unit costs**

Health and Social care resource use data were measured using data capture forms administered at baseline and all follow up periods. The total cost for each individual participant in the trial was calculated by multiplying their use of health and social care and societal resources by the associated unit cost. The mean cost per participant for the trial arm was then estimated. When it was not possible to obtain unit costs for the same price year, costs from earlier years were used and adjusted for inflation by applying the Hospital and Community Health Services (HCHS) pay and price index. Table 1 below lists the unit cost for all resources identified in the study.

**Supplementary Table 1: Unit costs**

| **Unit costs ASU** | | | | |
| --- | --- | --- | --- | --- |
| **Resource** | **Unit** | **Unit cost (£)** | **Source** | **Assumptions** |
| **GP** | visit | 42 | PSSRU 2022, Table 9.4.2, page 6 [1] | Per surgery consultation lasting 9.22 min, including direct care staff costs, with qualification costs |
| **NHS 24 (Number of calls)** | call | 8.61 | PPSRU 2022, Table 9.6, page 73[1] | Assume nursery-led triage, average duration is 6.56 min |
| **Paediatrician (children's doctor)** | hour | 143 | PSSRU 2022, Table 11.3.2[1] | Consultant, medical with qualification. Assume one hour visit. |
| **Dentist** | visit | 23 | PSSRU 2022, Table 9.7.1, page 70[1] | Assume 10 min as average duration. |
| **Hospital outpatient visit or day case** | visit | 235 | PSSRU 2022, Table 6.1, page 39[1] | Weighted average of all outpatient attendances |
| **Hospital A&E visit** | visit | 242 | NHS reference cost 2021/22[2] |  |
| **Speech and language therapist** | hour | 41 | PSSRU 2022, Table 11.1.2, page 89[1] |  |
| **Drugs, alcohol worker/addiction services** | hour | 88 | PSSRU 2022, Table 3.2.1[1] | assume face to face contact, with qualification |
| **Domestic violence services** | contact | 163.31 | UK Government[3] | legal aid, table 2(a) legal help |
| **Adult mental health services** | contact | 241 | PSSRU 2022, Table 2.1.1[1] | mental health care contacts |
| **Child & adolescent mental health services (CAMHS)** | contact | 126.15 | PSSRU 2017, 12.7, page 191[1] | assume face to face contact, hour |
| **Educational Psychologist** | contact | 46.90 | PSSRU 2014, pag. 156, table 8.8.3[1] |  |
| **Police Liaison Office** | hour | 34.04 | Police Scotland[4] |  |
| **Police call out due to incident or arrest** | hour | 46.38 | McDermid et al, 2013[5] |  |
| **Hospital inpatient (children)** | night | 614 | NHS reference cost 2021/22[2] | NHS reference cost 2021/22 main schedule, ‘Total HRGs’ tab; ‘Regular day or night admissions’ – Column Y. Average over all paediatric services (from record PC63A until PX57C). |
| **Hospital inpatient (adult)** | night | 376.8 | NHS reference cost 2021/22[2] | NHS reference cost 2021/22 main schedule, ‘Total HRGs’ tab; ‘Regular day or night admissions’ – Column Y. Consider all records except for neonatal/paediatric. |
| **days in residential or respite care** | day | 316.33 | NICE[6] |  |
| **nursery/childcare** | day | 53.9 | Web source | Cost per week of full time (50 hours) nursery for a child under 2 is 269.86, corresponding to 53.9/day |
|  |  |  |  |  |
| **Unit costs FACS** | | | | |
| **Resource** | **Unit** | **Unit Cost (£)** | **Source** | **Assumptions** |
| **Teacher school staff** | hour | 42 | Web source | Main grade, scale point 3 |
| **Child psychologist** | hour | 64 | PSSRU 2022, Table 11.1.2[1] | Assume Band 7 (clinical psychologist) |
| **Social care worker** | hour | 42 | PSSRU 2022, Table 10.1[1] | assume without qualifications |
| **GP/medical professional** | contact | 42 | PSSRU 2022, Table 9.4.2, page 66[1] | per surgery consultation lasting 9.22 min, including direct care staff costs, with qualification costs |
| **Health visitor** | hour | 56.69 | PSSRU 2015, Table 10.3[1] | assume cost per hour, with qualification |
| **Housing officer** | hour | 28.82 | Schneider et al. [7] |  |
| **Psychiatrist** | hour | 145 | PSSRU 2022, Table 11.3.2[1] | Consultant, medical, cost per working hour including qualifications |
| **Social worker** | hour | 42 | PSSRU 2022, Table 10.1[1] | assume without qualifications |
| **Social worker manager** | hour | 50 | PSSRU 2022, Table 10.1[1] | assume with qualifications |
| **Addiction worker** | hour | 88 | PSSRU 2022, Table 3.2.1[1] | assume face to face contact, with qualification |
|  |  |  |  |  |
| **Resource** | **Unit** | **Unit Cost (£)** | **Source** | **Assumptions** |
| **Health and social care staff (not NSPCC)** | | | | |
| **Addiction Worker** | hour | 88 | PSSRU 2022, page 24, Table 3.2.1[1] | assume with qualification and face-to-face contact |
| **Advocate (Social Care)** | hour | 42.12 | PSSRU 2021, page 79, table 6.9[1] | advocacy for children with additional/multiple needs |
| **Criminal Justice Worker** | hour | 21.47 | Web source |  |
| **Case Management Worker** | hour | 25.67 | Web source |  |
| **Intervention/support Worker** | hour | 25 | PSSRU 2022, page 82, Table 10.6.1[1] |  |
| **CAMHS/Mental Health Worker** | hour | 126.15 | PSSRU 2017, page 191[8] | generic multi-disciplinary CAMHS team, cost per hour per team member for face to face contact |
| **Children's Services Practitioner (Social Worker)** | hour | 42 | PSSRU 2022, page 77, Table 10.1[1] | assume without qualifications |
| **Clinical Psychologist** | hour | 64 | PSSRU 2022, Table 11.1.2[1] | Assume Band 7 (clinical psychologist) |
| **Contact Supervisor** | hour | 18.63 | Web source |  |
| **Dentist** | hour | 23 | PSSRU 2022, Table 9.7.1, page 70[1] | Assume 10 min as average duration. |
| **Disrupting exploitation Therapist** | hour | 23.29 | https://www.charityjob.co.uk/jobs/the-children-s-society/disrupting-exploitation-sexual-criminal-and-labour-exploitation-therapist-counsellor/578878?tsId=15 see calculation |  |
| **Doctor/Medical Professional** | contact | 42 | PSSRU 2022, Table 9.4.2, page 66[1] | per surgery consultation lasting 9.22 min, including direct care staff costs, with qualification costs |
| **Educational Welfare Officer** | hour | 26.58 | Unit Cost Calculation  https://nationalcareers.service.gov.uk/job-profiles/education-welfare-officer |  |
| **Family Nurse** | hour | 52 | PSSRU 2022, page 68, Table 9.3.1[1] | assume GP practice nurse with qualification |
| **Health Visitor** | hour | 56.69 | PSSRU 2015, Table 10.3[9] | assume cost per hour, with qualification |
| **Housing Officer** | hour | 25 | Schneider et al [7] |  |
| **Independent Reviewing Officer** | hour | 51.92 | Web source |  |
| **Interpreter** | hour | 20.62 | Web source |  |
| **Lawyer** | hour | 135 | Web source | Legal aid (maximum) |
| **Nursery Key Worker** | hour | 21.38 | Web source |  |
| **Paediatrician** | hour | 145 | PSSRU 2022, page 101, Table 11.3.2[1] | Medical consultant with qualification |
| **Respite Carer** | hour | 0 | Web source |  |
| **Senior Addictions Worker** | hour | 88 | PSSRU 2022, Table 3.2.1 [1] | assume face to face contact, with qualification |
| **Social care worker** | hour | 42 | PSSRU 2022, Table 10.1 [1] | assume without qualifications |
| **Social Work Assistant** | hour | 33 | PSSRU 2022, page 82, Table 10.2 [1] |  |
| **Social Work Manager** | hour | 50 | PSSRU 2022, Table 10.1 [1] | assume with qualifications |
| **Social Worker** | hour | 42 | PSSRU 2022, Table 10.1 [1] | assume without qualifications |
| **Speech and language therapist** | hour | 41 | PSSRU 2022, Table 11.1.2, page 89 [1] |  |
| **Supervising Social Worker** | hour | 50 | PSSRU 2022, Table 10.1 [1] |  |
| **Support Worker** | hour | 25 | PSSRU 2022, page 82, Table 10.6 [1] |  |
| **Welfare Rights Officer** | hour | 27.3 | Unit Cost Calculation |  |
| **Women's Aid Children's Worker** |  | 19.1 | Web source | Support workers |
| **Probation worker** | hour | 29.14 | Web source |  |
| **NSPCC Staff** | | | | |
| **NSPCC CSP Clinical Psychologist** | hour | 64 | PSSRU 2022, Table 11.1.2 [1] | Assume Band 7 (clinical psychologist) |
| **NSPCC CSP level 3 Clinical Director (clinical psychologist)** | hour | 64 | PSSRU 2022, Table 11.1.2 [1] |  |
| **NSPCC CSP level 3 Clinical Director consultant psychiatrist** | hour | 145 | PSSRU 2022, Table 11.3.2 [1] |  |
| **NSPCC CSP level 3 Consultant Psychiatrist** | hour | 145 | PSSRU 2022, Table 11.3.2 [1] | Consultant, medical, cost per working hour including qualifications |
| **NSPCC CSP level 3 Principal Clinical Psychologist** | hour | 86 | PSSRU 2022, page 94, table 11.1.2 [1] | cost per working hour, Band 8b |
| **NSPCC CSP level 3 Social Worker** | hour | 42 | PSSRU 2022, Table 10.1 [1] | assume without qualifications |
| **NSPCC Children services practitioner level 3** | hour | 42 | PSSRU 2022, page 77, Table 10.1 [1] | assume without qualifications |
| **NSPCC Clinical Lead Practitioner (clinical psychologist)** | hour | 64 | PSSRU 2022, Table 11.1.2 [1] |  |
| **NSPCC Clinical Lead Practitioner psychiatrist** | hour | 145 | PSSRU 2022, Table 11.3.2 [1] |  |
| **NSPCC Clinical Practitioner** | hour | 37.31 | Web source |  |
| **NSPCC Clinical Principle Psychologist** | hour | 86 | PSSRU 2022, page 94, table 11.1.2 [1] | cost per working hour, Band 8b |
| **NSPCC Clinical Psychologist** | hour | 64 | PSSRU 2022, Table 11.1.2 [1] | Assume Band 7 (clinical psychologist) |
| **NSPCC Consultant Social Worker** | hour | 42 | PSSRU 2022, Table 10.1[1] | assume without qualifications |
| **NSPCC associate clinical director** | hour | 86 | PSSRU 2022, page 94, table 11.1.2, band 8b [1] |  |
| **NSPCC children service practitioner** | hour | 42 | PSSRU 2022, page 77, Table 10.1[1] | assume without qualifications |
| **NSPCC family liaison worker** | hour | 32.36/hr | Web source |  |

**Supplementary Appendix S2**

Table 2 below shows the percentage of missing data, by intervention arm, for baseline characteristics and total cost and outcomes (PedSQL scores; CHU9D derived scores; SDQ scores), at each time point. When considering PedsQL, in both arms the percentage of missingness goes from 17.1% at baseline to 28.5% in the last follow-up. The SDQ shows a rate of missingness close to 60% at baseline, that decreases to 20% at visit 3. A higher completion rate for ASU is observed at baseline (43.3%), as compared to last follow-up (30%). The table shows a similar pattern of missing data between arms, but a different percentage of missing data between time points. A proportion of missing data which differs across timepoints suggests that data are unlikely to be missing completely at random (MCAR), making complete-case analysis biased.

We further investigated the mechanism of missingness, exploring whether the probability of observing missing costs and outcomes is significantly associated with both baseline characteristics and, with previously observed values of costs and outcomes. Results (not reported, but available upon request) rule out the possibility that data are MCAR and covariate-dependent-missing (CD-MAR). The reasons discussed above, and, the large proportion of data lost for the complete-case analysis strengthen the rationale for using the multiple imputation data sets in the base case. The cost-effectiveness results using the complete case are presented for comparison.

**Supplementary Table 2: Missing data**

|  | **Combined (NIM +SAU)** | | **NIM** | | **SAU** | |
| --- | --- | --- | --- | --- | --- | --- |
|  | No. missing values | % over total | No. missing values | % over total NIM | No. missing values | % over total SAU |
| Variable |  |  |  |  |  |  |
| CHU9D-visit1 | 75 | **17.1%** | 36 | **16.1%** | 39 | **18.1%** |
| CHU9D-visit2 | 104 | **23.7%** | 57 | **25.6%** | 47 | **21.8%** |
| CHU9D-visit3 | 125 | **28.5%** | 61 | **27.4%** | 64 | **29.6%** |
| PedsQL-visit1 | 75 | **17.1%** | 36 | **16.1%** | 39 | **18.1%** |
| PedsQL-visit2 | 104 | **23.7%** | 57 | **25.6%** | 47 | **21.8%** |
| PedsQL-visit3 | 125 | **28.5%** | 61 | **27.4%** | 64 | **29.6%** |
| SDQ-visit1 | 260 | **59.2%** | 129 | **57.8%** | 131 | **60.6%** |
| SDQ-visit2 | 207 | **47.2%** | 106 | **47.5%** | 101 | **46.8%** |
| SDQ-visit3 | 88 | **20.0%** | 39 | **17.5%** | 49 | **22.7%** |
| ASU - visit1 | 190 | **43.3%** | 91 | **40.8%** | 99 | **45.8%** |
| ASU - visit2 | 142 | **32.3%** | 77 | **34.5%** | 65 | **30.1%** |
| ASU - visit3 | 133 | **30.3%** | 61 | **27.4%** | 72 | **33.3%** |
| Age | 0 | **0.0%** | 0 | **0.0%** | 0 | **0.0%** |
| Gender | 5 | **1.1%** | 2 | **0.9%** | 3 | **1.4%** |
| site | 0 | **0.0%** | 0 | **0.0%** | 0 | **0.0%** |
| Family type | 0 | **0.0%** | 0 | **0.0%** | 0 | **0.0%** |

Note: the number of missing values in the health economics analysis differs from the number of missing in the main analysis. Indeed, in the health economics analysis the percentage of missing data has been calculated over the full sample size (439 children randomised to BEST). In the main analysis, the percentage of missing data at each time point has been calculated over the number of expected questionnaires to be filled out at a visit, based on visit attendance, age of subjects at visit, and whether a subject has reached a visit without withdrawing first. Children with no attendance at a visit are therefore not counted as part of expected completion.

**Supplementary Appendix S3: summary statistics**

***Additional service use***

Table 3 presents the mean difference in total additional service use cost accrued throughout the trial duration between trial arm from the multiply imputed dataset. The table shows *NHS&PSS costs* (including healthcare costs and costs related to usage of social services); *Public Sector costs* (including additional broader costs, such as contacts with the police and admissions to residential or respite care); *Societal costs* (including the cost of childcare in addition to *Public Sector costs*). The table shows no statistically significant differences between arms, when considering all three cost categories.

Table 4 and Table 5 show summary statistics for cost categories, per participant per arm and time point, for the complete case analysis and the multiply imputed dataset.

**Supplementary Table 3: Total cost (additional service use) by sector and by arm**

|  | **NIM** | **SAU** | **Difference** |
| --- | --- | --- | --- |
|  | Mean (sd) | Mean (sd) |  |
|  |  |  |  |
| **Total NHS & PSS Costs (£)** | **2898** | **3193** | -295 |
|  | (4840) | (5562) | (528) |
|  |  |  |  |
| **Total Public sector costs (£)** | **3206** | **3054** | 152 |
|  | (5117) | (5293) | (556) |
|  |  |  |  |
| **Total Societal costs (£)** | **11767** | **12794** | -1027 |
|  | (10090) | (11919) | (1501) |
|  |  |  |  |

Note: summary statistics on multiply-imputed dataset; NHSPSS costs include costs referring to the usage of NHS and Social services; Public sector costs include contacts with the Police and admission in residential and respite care in addition to NHSPSS costs. Societal costs include cost of childcare in addition to public sector costs.

**Table 4: Cost categories, by trial arm and time point, multiply-imputed dataset**

| Trial arm | **Variable** | **Mean** | SD | min | max | N |
| --- | --- | --- | --- | --- | --- | --- |
|  |  |  |  |  |  |  |
| SAU | **NHSPSS_cost_v1** | **802** | 2196 | 0 | 29356 | 216 |
|  | **NHSPSS_cost_v2** | **963** | 1504 | 0 | 10696 | 216 |
|  | **NHSPSS_cost_v3** | **2278** | 4841 | 0 | 48336 | 216 |
|  |  |  |  |  |  |  |
| NIM | **NHSPSS_cost_v1** | **628** | 1671 | 0 | 19034 | 223 |
|  | **NHSPSS_cost_v2** | **999** | 2082 | 0 | 24780 | 223 |
|  | **NHSPSS_cost_v3** | **1942** | 3934 | 0 | 28227 | 223 |
|  |  |  |  |  |  |  |
| SAU | **PSect_cost_v1** | **996** | 2303 | 0 | 29356 | 216 |
|  | **PSect_cost_v2** | **1118** | 2408 | 0 | 29356 | 216 |
|  | **PSect_cost_v3** | **1982** | 3545 | 0 | 29356 | 216 |
|  |  |  |  |  |  |  |
| NIM | **PSect_cost_v1** | **1386** | 3438 | 0 | 38000 | 223 |
|  | **PSect_cost_v2** | **1363** | 2378 | 0 | 19050 | 223 |
|  | **PSect_cost_v3** | **1891** | 3600 | 0 | 21543 | 223 |
|  |  |  |  |  |  |  |
| SAU | **Societal_cost_v1** | **2127** | 3029 | 0 | 29356 | 216 |
|  | **Societal_cost_v2** | **3095** | 4333 | 0 | 33968 | 216 |
|  | **Societal_cost_v3** | **9891** | 10309 | 0 | 40692 | 216 |
|  |  |  |  |  |  |  |
| NIM | **Societal_cost_v1** | **2340** | 3969 | 0 | 38041 | 223 |
|  | **Societal_cost_v2** | **3097** | 3821 | 0 | 19520 | 223 |
|  | **Societal_cost_v3** | **8847** | 8884 | 0 | 36254 | 223 |
|  |  |  |  |  |  |  |

*Note: v1: visit 1 (baseline); v2: visit 2 (1 year after baseline); v3: visit3 (2.5 years after baseline)*

**Supplementary Table 5 : Cost categories, by trial arm and time point, complete-case analysis**

| **Trial arm** | **Variable** | Mean | N | SD | Min | Max |
| --- | --- | --- | --- | --- | --- | --- |
|  |  |  |  |  |  |  |
|  |  |  |  |  |  |  |
| **SAU** | **NHSPSS_cost_v1** | **920** | 117 | 2845 | 0 | 29356 |
|  | **NHSPSS_cost_v2** | **984** | 151 | 1571 | 0 | 10696 |
|  | **NHSPSS_cost_v3** | **2474** | 144 | 5483 | 0 | 48336 |
|  |  |  |  |  |  |  |
| **NIM** | **NHSPSS_cost_v1** | **719** | 132 | 1955 | 0 | 19034 |
|  | **NHSPSS_cost_v2** | **1046** | 146 | 2263 | 0 | 24780 |
|  | **NHSPSS_cost_v3** | **1966** | 162 | 3984 | 0 | 28227 |
|  |  |  |  |  |  |  |
| **SAU** | **PSect_cost_v1** | **1115** | 117 | 2926 | 0 | 29356 |
|  | **PSect_cost_v2** | **1291** | 151 | 1872 | 0 | 11645 |
|  | **PSect_cost_v3** | **3447** | 144 | 6534 | 0 | 59723 |
|  |  |  |  |  |  |  |
| **NIM** | **PSect_cost_v1** | **1468** | 132 | 3929 | 0 | 37956 |
|  | **PSect_cost_v2** | **1679** | 146 | 2777 | 0 | 24860 |
|  | **PSect_cost_v3** | **2929** | 162 | 4681 | 0 | 29176 |
|  |  |  |  |  |  |  |
| **SAU** | **Societal_cost_v1** | **1988** | 117 | 3429 | 0 | 29356 |
|  | **Societal_cost_v2** | **2592** | 151 | 3491 | 0 | 21479 |
|  | **Societal_cost_v3** | **8535** | 144 | 11336 | 0 | 75489 |
|  |  |  |  |  |  |  |
| **NIM** | **Societal_cost_v1** | **2123** | 132 | 4343 | 0 | 37956 |
|  | **Societal_cost_v2** | **2873** | 146 | 3985 | 42 | 29711 |
|  | **Societal_cost_v3** | **7317** | 162 | 8834 | 0 | 36879 |
|  |  |  |  |  |  |  |

*Note: v1: visit 1 (baseline); v2: visit 2 (1 year after baseline); v3: visit3 (2.5 years after baseline)*

***Outcomes***

Table 6 summarizes the quality of life scores at each follow-up point (multiply-imputed dataset), calculated from mapping the PedsQl scores to the Child Health Utility Instrument (CHU-9D) to generate a utility value for a QALY [10]. Table 4 also reports overall QALYs, calculated over the trial period using the area under the curve method[11]. CHU-9D scores are not statistically significantly higher in the NIM arm at baseline and Time 1, at Time 3 the SAU arm also shows a not statistically significant higher score than the NIM arm. Similarly, as shown in Table 6 there is no statistically significant differences between arms in terms of total QALYs.

While imputation was undertaken using transformed CHU-9D scores, PedsQL scores for the available-case analysis are reported in Supplementary Table 7. Supplementary Table 8 and 9 also reports descriptive statistics on the SDQ total score, for the available-case analysis and the multiply-imputed dataset.

**Supplementary Table 6: Mean utility scores and QALY, per arm**

| **CHU-9D Mean utility scores, by trial arm and time point** | | | |
| --- | --- | --- | --- |
|  | **NIM** | **SAU** |  |
| **Time point** | **Mean (sd)** | **Mean (sd)** | **Difference (sd)** |
|  |  |  |  |
| baseline | **0.927** | **0.9259** | **0.0011** |
|  | -0.0398 | -0.0414 | -0.0041 |
| Time 1 (1 year after baseline) | **0.9258** | **0.9256** | **0.0002** |
|  | -0.0353 | -0.0396 | -0.0039 |
| Time 2 (2.5 years after baseline) | **0.9179** | **0.9217** | **-0.0038** |
|  | -0.0428 | -0.0404 | (-0.0035) |
| **Total QALY, by trial arm** | | | |
|  | **NIM** | **SAU** |  |
|  | Mean (sd) | Mean (sd) | Difference (sd) |
|  |  |  |  |
| Total QALY | **2.2888** | **2.2907** | **-0.0019** |
|  | -0.075 | -0.0853 | -0.0081 |

**Supplementary Table 7: Total PedsQL scores, by trial arm and time point, available-cases analysis**

| **Trial arm** |  | **PedsQL total score_v1** | **PedsQL total score_v2** | **PedsQL total score_v3** |
| --- | --- | --- | --- | --- |
|  |  |  |  |  |
| **SAU** | **mean** | **87.44958** | **87.97438** | **87.30204** |
|  | sd | 12.19073 | 12.01999 | 12.39551 |
|  | N | 177 | 169 | 152 |
|  | min | 44.04762 | 28.57143 | 41.66667 |
|  | max | 100 | 100 | 100 |
|  |  |  |  |  |
| **NIM** | **mean** | **87.33546** | **88.15489** | **87.19839** |
|  | sd | 11.94717 | 10.75092 | 12.28183 |
|  | N | 187 | 166 | 162 |
|  | min | 35.71429 | 43.47826 | 36.95652 |
|  | max | 100 | 100 | 100 |
|  |  |  |  |  |
| **Total** | **mean** | **87.39095** | **88.06383** | **87.24857** |
|  | sd | 12.0497 | 11.3921 | 12.31737 |
|  | N | 364 | 335 | 314 |
|  | min | 35.71429 | 28.57143 | 36.95652 |
|  | max | 100 | 100 | 100 |

*Note: v1: visit 1 (baseline); v2: visit 2 (1 year after baseline); v3: visit3 (2.5 years after baseline)*

**Supplementary Table 8: SDQ total scores, by trial arm and time point, available-cases analysis**

| **Treatment arm** |  | SDQ TOTAL SCORE_v1 | SDQ TOTAL SCORE_v2 | SDQ TOTAL SCORE_v3 |
| --- | --- | --- | --- | --- |
|  |  |  |  |  |
| **SAU** | **Mean** | **14.02** | **12.55** | **11.08** |
|  | SD | 8.13 | 6.87 | 7.18 |
|  | Min | 0 | 1 | 0 |
|  | Max | 33 | 34 | 30 |
|  | N | 85 | 115 | 167 |
|  |  |  |  |  |
| **NIM** | **Mean** | **11.83** | **11.74** | **11.49** |
|  | SD | 7.96 | 7.02 | 7.60 |
|  | Min | 0 | 0 | 0 |
|  | Max | 37 | 30 | 31 |
|  | N | 94 | 117 | 184 |

**Supplementary Table 9: SDQ total scores, by trial arm and time point, multiply -imputed dataset**

|  |  | SDQ TOTAL SCORE_v1 | SDQ TOTAL SCORE_v2 | SDQ TOTAL SCORE_v3 |
| --- | --- | --- | --- | --- |
|  |  |  |  |  |
| **SAU** | **Mean** | **12.68** | **11.27** | **10.95** |
|  | SD | 7.75 | 6.60 | 7.14 |
|  | Min | 0 | 1 | 0 |
|  | Max | 33 | 34 | 30 |
|  | N | 216 | 216 | 216 |
|  |  |  |  |  |
|  | **Mean** | **11.33** | **11.79** | **11.50** |
| **NIM** | SD | 7.55 | 6.95 | 7.56 |
|  | Min | 0 | 0 | 0 |
|  | Max | 37 | 30 | 31 |
|  | N | 223 | 223 | 223 |

**Supplementary Appendix S4: CUA: Additional results**

**Supplementary Table 10: CUA results, complete-case analysis**

| **CUA** |  |  |  |  |  |  |
| --- | --- | --- | --- | --- | --- | --- |
|  | **NIM** | **SAU** | **Difference** | **Boostrapped CI** |  | **ICER** |
| **QALY** | 2.290 | 2.297 | **-0.0072** | *-0.0310* | *0.0093* | SAU dominates |
| **Cost: cost of intervention/control** | 10344 | 5503 | **4841** | *1960* | *8475* | SAU dominates |
| **Cost: cost of intervention/control + ASU (Public sector perspective)** | 14094 | 9970 | **4124** | *632* | *8059* | SAU dominates |
| **Cost: cost of intervention/control + ASU (NHS&PSS perspective)** | 12568 | 8451 | **4117** | *-1606* | *9841* | SAU dominates |
| **Cost: cost of intervention/control + ASU (Societal perspective)** | 21289 | 17473 | **3816** | *1062* | *8261* | SAU dominates |

**Supplementary Figure 1 Cost-effectiveness plane, Multiple-imputation, base-case analysis**

***Supplementary Appendix S5: Complier Average Causal Effect (CACE) analysis***

We found that 66% of those randomised to the NIM intervention received an assessment and/or a treatment. Specifically, families of 106 children were offered and engaged in both assessment and treatment; 35 families were offered and engaged in full assessment but did not engage in the treatment that was offered; 7 families were offered and engaged in a partial assessment. The remaining 34% of families fall in the ‘non-compliance’ category (no-engagers; not consented to treatment; received a consultation to decide whether an assessment was still required). Results confirm the main ITT analysis, with SAU dominating NIM (Supplementary Table 11).

**Supplementary Table 7: CACE analysis**

| **Cost-utility analysis** |  |  |  |  |  |
| --- | --- | --- | --- | --- | --- |
|  |  | **NIM** | **SAU** | **Difference** | **ICER** |
| **Base case analysis** | **QALY** | 2.2881 | 2.289 | **-0.0009** | SAU dominates |
|  | **NHS&PSS perspective Cost (£): cost of intervention/control** | 10315 | 5845 | **4470** | SAU dominates |
| **Scenario I** | **QALY** | 2.2875 | 2.2893 | **-0.0019** | SAU dominates |
|  | **Scenario 1: NHS&PSS perspective) Cost (£): cost of intervention/control + ASU )** | 12894 | 9179 | **3715** | SAU dominates |
| **Scenario II** | **QALY** | 2.2881 | 2.289 | **-0.0009** | SAU dominates |
|  | **Public sector perspective Cost £): cost of intervention/control + ASU** | 13465 | 8908.563 | **4557** | SAU dominates |
| **Scenario III** | **QALY** | 2.2881 | 2.289 | **-0.0009** | SAU dominates |
|  | **Scenario 3: Cost (£): cost of intervention/control + ASU** | 21924 | 18436 | **3488** | SAU dominates |
|  |  |  |  |  |  |
| **Cost-effectiveness analysis** | |  |  |  |  |
|  |  | **NIM** | **SAU** | **Difference** | **ICER** |
| **Base case analysis** | **SDQ T3- SDQ T1** | -1.0244 | -0.4997 | **0.5248** | SAU dominates |
|  | **Cost: cost of intervention/control** | 10341 | 5832 | **4509** | SAU dominates |

****ASU= additional service use***

1. Jones KC, Weatherly H, Birch S, et al. Unit costs of health and social care 2022 manual. 2022

2. Department of Health. NHS Refererence Costs: Financial year 2020/2021. Secondary NHS Refererence Costs: Financial year 2020/2021 2020.

3. UK Government. Secondary 2010. <https://assets.publishing.service.gov.uk/government/uploads/system/uploads/attachment_data/file/308903/LAA-2010-payment-annex-2.pdf>.

4. Police Scotland. Secondary 2010. <https://www.scotland.police.uk/about-us/finance/pay-and-grading-structure/>.

5. McDermid S, Holmes L. The cost effectiveness of action for children’s intensive family support services. Action for Children Breakfast Briefing-Troubled Families, Where Next 2013

6. NICE. Service models guidance: individuals with intellectual disabilities and behaviour that challenges. Secondary Service models guidance: individuals with intellectual disabilities and behaviour that challenges. <https://www.nice.org.uk/guidance/ng93/evidence/appendix-c3-economic-model-for-respite-care-pdf-4788958434>.

7. Schneider J, Boyce M, Johnson R, et al. Impact of supported employment on service costs and income of people with mental health needs. Journal of Mental Health 2009;**18**(6):533-42

8. Curtis LB, A. . Unit Costs of Health and Social Care 2017, Personal Social Services Research Unit, University of Kent, Canterbury

2017

9. Curtis LB, A. . Unit Costs of Health and Social Care 2015, Personal Social Services Research Unit, University of Kent, Canterbury. 2015

10. Kelly CB, Soley-Bori M, Lingam R, et al. Mapping PedsQL™ scores to CHU9D utility weights for children with chronic conditions in a multi-ethnic and deprived metropolitan population. Quality of Life Research 2023:1-15

11. Manca A, Hawkins N, Sculpher MJ. Estimating mean QALYs in trial‐based cost‐effectiveness analysis: the importance of controlling for baseline utility. Health economics 2005;**14**(5):487-96
